# Perceiving the Invisible Threat: Are Allergic Individuals Aware of the Health Risks of Micro- and Nanoplastics?

**DOI:** 10.3390/children13040470

**Published:** 2026-03-28

**Authors:** Ana Kujavec, Manuela Oroz, Jan Pantlik, Ivana Banić, Sandra Mijač, Ana Vukić, Petra Anić, Ana-Marija Genc, Antonija Piškor, Maja Šutić, Marcel Lipej, Željka Vlašić Lončarić, Milan Jurić, Ivana Marić, Vlatka Drinković, Tin Kušan, Rajka Lulić Jurjević, Mirjana Turkalj

**Affiliations:** 1 Department of Medical Research, Srebrnjak Children’s Hospital, HR-10000 Zagreb, Croatia; 2 Department of Innovative Diagnostics, Srebrnjak Children’s Hospital, HR-10000 Zagreb, Croatia; 3 IT Department, Srebrnjak Children’s Hospital, HR-10000 Zagreb, Croatia; 4 Department of Pulmonology, Srebrnjak Children’s Hospital, HR-10000 Zagreb, Croatia; 5 Department of Allergy and Clinical Immunology, Srebrnjak Children’s Hospital, HR-10000 Zagreb, Croatia; 6 Department of Cardiology, Srebrnjak Children’s Hospital, HR-10000 Zagreb, Croatia; 7 Faculty of Medicine, J.J. Strossmayer University of Osijek, HR-31000 Osijek, Croatia; 8 Faculty of Medicine, Catholic University of Croatia, HR-10000 Zagreb, Croatia

**Keywords:** microplastics, nanoplastics, public perception, allergies, education, urbanicity, health risk awareness

## Abstract

**Highlights:**

**What are the main findings?**

- Allergic participants demonstrated higher awareness and greater concern about micro- and nanoplastics (MNPs) than non-allergic peers.
- Education level was the strongest predictor of MNP awareness, while urbanicity affected terminology recognition but not risk perception.

**What are the implications of the main findings?**

- MNP risk communication should account for differences related to allergic vulnerability and educational background.
- Focused educational strategies are needed to translate awareness into health-protective behaviors and policy engagement.

**Abstract:**

**Background:** Micro- and nanoplastics (MNPs) are widespread environmental contaminants with growing evidence linking them to adverse health effects, including progression and worsening of allergic diseases. As allergies are rapidly increasing among youth (affecting almost 30% of children), this demographic represents a vulnerable population facing emerging environmental threats. Since no prior study has investigated MNP risks perceptions in an allergic population, this study aimed to assess public awareness and risk perception of MNP in Croatian youth, focusing on the influence of urbanicity, education, and allergy status. **Methods:** A total of 1155 participants (aged 6–18 years) were recruited from three Croatian regions as part of the EU Horizon 2020 IMPTOX and the Horizon Europe EDIAQI studies. Allergy status was determined via skin prick tests (SPT), and standardized questionnaires were used to collect data on MNP awareness and perception. **Results:** Awareness was significantly higher among allergic individuals (89.5% vs. non-allergic 79%, FDR *p* value= 0.036) and those with university-level education (88.3% vs. elementary 63.3%, FDR *p* value = 0.050). Allergic participants were also more concerned about food contamination by MNPs (87.7%) compared to non-allergic individuals (79.2%), FDR *p* value = 0.005). Media and social media were the primary sources of information regarding MNPs (FDR *p* value = 0.026). **Conclusions:** Education and allergy status are the strongest predictors of MNP awareness and related risk perceptions in the Croatian population. Targeted public health communication and educational strategies are needed to translate basic awareness into informed behavioral and policy engagement.

## 1. Introduction

The current era is characterized by the pervasive presence of plastics, a synthetic material that has revolutionized countless aspects of our daily lives. Yet the very durability that makes plastics so practical also contributes to a growing environmental challenge. As these materials degrade, they produce microplastic (MPs, <5 mm) and even smaller nanoplastic particles (NPs, <100 nm) [1]. These microscopic particles are now abundant in marine, freshwater, terrestrial, and atmospheric ecosystems, making them an omnipresent pollutant [2,3,4,5]. Alarmingly, these particles have also been detected in human samples, including blood, stool, placenta, breast milk and ovarian follicular fluid, raising concerns about potential health impacts [6,7,8,9,10]. Micro- and nanoplastic particles (MNPs) originate from diverse sources such as the breakdown of larger plastic items, industrial processes, vehicle tires, city dust, microbeads in cosmetic products, and synthetic fibers from textiles [11,12]. They can enter the human body through different routes such as inhalation (contaminated air), ingestion (salt, seafood, bottled beverages) and dermal contact (personal care products) [13,14]. Studies have shown that MNPs can trigger inflammation, apoptosis, oxidative stress, and disruptions in key biological processes, including immune system dysfunction [15,16,17,18,19,20,21,22]. Accumulation of MNPs may also contribute to long-term health issues, such as development of cancer, metabolic and autoimmune disorders, reproductive problems, and neurodevelopmental conditions, through aforementioned pathophysiological mechanisms [15,23,24,25,26]. The severity of these effects varies depending on particle size, composition, route and duration of exposure. Despite growing evidence, a number of gaps in knowledge prevent an all-encompassing risk assessment of these effects on human health. Firstly, MNPs may pose a health hazard themselves, being directly toxic as shown in laboratory settings, but they are also more commonly associated with other biotic and abiotic agents, absorbing and carrying other components (such as heavy metals, certain chemicals, some of which are known endocrine disruptors, and microorganisms), which makes these particles even more harmful. Of particular concern is their potential role in immune system modulation, with emerging evidence suggesting MNPs may act as adjuvants, exacerbating allergic responses by disrupting epithelial barriers or carrying adsorbed allergens (e.g., pollen, metals) into tissues [16,27]. Although advancements have been made in estimating the health risks of MNPs [28], addressing such complex mixtures remains difficult. In addition, the levels of these toxic effects in humans are unknown, and studies that estimate these levels are still lacking. Moreover, methodological limitations in the characterization of smaller particles (especially NPs), which may have more profound health effects since they are able to penetrate tissues deeper than larger particles, also contribute to a great deal of uncertainties around MNPs and human health.

Public concern regarding plastic pollution is growing, driven by increasing media attention and environmental awareness. While early microplastic research has traditionally focused on their environmental distribution and ecological impacts, recent work has increasingly shifted toward understanding potential impacts on human health [29]. Although several studies have explored public awareness and social perceptions of MNPs across different groups, none have specifically addressed vulnerable populations such as children, particularly those with allergic conditions, while simultaneously accounting for key demographic factors such as residential area and education level [2,3,4,5,6,7,8,9,10,11,12,13,14,15,16,17,18,19,20,21,22,23,24,25,26,27,28,29,30,31,32,33,34,35,36,37,38,39]. This represents a critical gap, as accumulating evidence suggests that MNPs may negatively affect immune responses and exacerbate allergic diseases. Allergies are the most common chronic condition in Europe, with a particularly high prevalence among younger populations, affecting over 30% of children and adolescents [40,41]. With sensitization rates continuing to rise, the burden of allergic diseases is expected to further increase in the coming years. Against this background, exploring perceptions of MNP-related health risks among allergic individuals offers critical insights into how vulnerable groups engage with emerging environmental threats.

Moreover, social perception studies in this area are highly relevant, as perceptions influence behavioral change, public support for policy interventions, and the effectiveness of health risk communication [42,43]. In this context, our study aims to examine perceptions and awareness of MNP-related health risks within the Croatian population, with a focus on demographic characteristics, place of residence, education level and allergy status. To the best of our knowledge, this is the first study assessing consumer perception and awareness of MNPs in patients with chronic diseases, namely allergic conditions.

## 2. Methodology

This research comprised a single-center (lead by the Srebrnjak Children’s Hospital in Zagreb, Croatia), cross-sectional, observational study. Pediatric participants, aged 6–18 years, were included through a stratified sampling approach based on sex, age group, residential area, and differential exposure and susceptibility to allergy (allergic vs. healthy non-allergic subjects, schoolchildren). The participants were further divided into groups according to their microgeographical region of origin: the capital city of Zagreb and its surroundings, the eastern continental regions of Slavonia and the Mediterranean region of Dalmatia. These regions were specifically chosen due to their differing levels of exposure to allergens and pollutants, as well as variations in lifestyle and dietary habits. This work was conducted as part of the Horizon 2020 IMPTOX (An innovative analytical platform to investigate the effect and toxicity of micro- and nanoplastics combined with environmental contaminants on the risk of allergic disease in preclinical and clinical studies, grant agreement number: 965173) and the Horizon Europe EDIAQI (Evidence-driven indoor air quality improvement, grant agreement number: 101057497) research projects. 

### 2.1. Study Population

Participants were recruited in primary and high schools in Croatia, following a dedicated meeting with the school administration and parents/caregivers, as well as older children. Informed written consent was obtained from the children’s caregivers for all participants. The study protocols were approved by the local Ethics Committee at the Srebrnjak Children’s Hospital on 19 October 2021 (CLASS: 100-02/21-01, Rec. No.: 04-930/3-21) and on 1 February 2023 (CLASS: 100-02/23-01), Rec. No.: 04-100/3-23), Supplementary Materials (Figures S1–S4). The studies were registered at Clinicaltrials.gov (ID: NCT05177744 and ID: NCT05992389).

From October 2022 to April 2024, a total of 1155 participants (aged 6–18 years, both sexes) were recruited, whose parents/caregivers signed an informed consent after receiving both written and oral information about the study. Inclusion and exclusion criteria for participation were assessed by a pediatric allergy specialist with over 20 years of expertise in the field. Inclusion criteria included the following: school-aged children, allergic and non-allergic, otherwise healthy. Additionally, for the allergic/sensitized group of children, inclusion criteria involved proven sensitization to at least one allergen and/or a diagnosis of one or more allergic diseases (allergic rhinoconjuctivitis, allergic asthma, food allergy and/or atopic dermatitis). Exclusion criteria included being previously diagnosed with congenital and other chronic illnesses which may affect the results of this study, such as systemic mastocytosis and severe metabolic disorders. Lastly, children were excluded from participation and biomaterial collection if they currently had a fever above 38.5 °C or a known acute infection.

### 2.2. Assessments and Data Collection

Upon recruitment, all participants underwent a skin prick test (SPT) using a standard palette of food and inhaled allergens (detailed in Supplementary Materials Table S1). Standardized SPT controls were obtained from Diater Laboratorio de Diagnóstico y Aplicaciones Terapéuticas, Madrid, Spain. Allergen extracts were freshly prepared by macerating fresh or frozen food allergen with 1 mL of saline solution. A single drop of each solution was applied to the volar surface of the forearm, followed by a skin prick using a sterile lancet. Histamine hydrochloride (1 mg/mL) served as the positive control, while saline solution was used as the negative control. Reactions were measured 15 min post-application [44].

Additionally, participants whose caregivers provided consent also underwent blood sampling. Peripheral whole-blood samples were collected via venipuncture into vacutainers containing a gel clot activator. Following collection, vacutainers with blood samples were centrifuged at 3000× *g* for 10 min for serum separation. Serum samples were then stored at −20 °C until further analysis.

All diagnostic procedures and assessments were done by the same personnel (from the Srebrnjak Children’s Hospital) to avoid inter-operator variability.

### 2.3. Questionnaires and Socioeconomic Data Collection

Data on personal and family history of allergic diseases, socioeconomic data, lifestyle and dietary habits, as well as data on participant awareness and perception of MNPs, was collected using existing questionnaires (Supplementary Table S2) [45,46,47]. Respondents aged 12 years or older were asked to complete the questionnaire themselves, while for children under the age of 12, it was completed by their parents or caregivers. Socioeconomic data, including the highest attained education level, were collected from the parents/caregivers, while health-related data (e.g., allergy diagnosis) pertained to their children.

### 2.4. Data Processing and Statistical Analysis

Features containing string notations were numerically encoded. Allergic sensitization data were expressed as a sensitization index, calculated as the ratio of the average urtica wheel diameter of the tested allergen to the urtica wheel diameter of histamine (positive control) in the SPT (sensitization index). A sensitization index of ≥0.6 was considered a positive reaction to an allergen. Additionally, certain features of allergic sensitization (e.g., polysensitization) and morbidity (allergic diseases) were converted into binary or ordinal features (e.g., elevated/normal, low/normal/high, yes/no). The Kolmogorov–Smirnov test was used to assess the normality of data distribution. Differences in the prevalence of allergic sensitization and allergic diseases were analyzed using the χ^2^ test as crude values.

A multivariate regression analysis was performed to assess the association between selected sociodemographic, environmental, and behavioral factors and the outcome of interest, adjusted for common confounders (age and sex of the participants and socioeconomic status—SES). Prior to analysis, variables with multiple categories and sparse observations were recoded by collapsing conceptually related categories to ensure adequate sample size per level and improve model stability. Prior to modeling, multiple imputation was performed on each outcome–predictor set using a custom-built function that leverages the mice package of the R Project for Statistical Computing (version R-4.4.3 for Windows). The imputation was tailored according to variable type: predictive mean matching was applied for continuous variables, logistic regression for binary variables, multinomial logistic regression for nominal categorical variables, and proportional odds models for ordered factors. A total of 10 imputed datasets were generated with 10 iterations each to ensure convergence and stability of the imputation process. Multivariable regression models were fitted separately within each imputed dataset. Reference categories were defined a priori based on meaningful baseline groups to facilitate interpretation of odds ratios. Final model estimates—including odds ratios, standard errors, test statistics, degrees of freedom, *p*-values, and confidence intervals—were derived from the pooled logistic regression results using Rubin’s rules, ensuring valid inference across all multiply imputed datasets. Model performance was evaluated using accuracy, area under the receiver operating characteristic curve (AUC), precision, recall, and F1-score for binary outcomes, while accuracy was reported for multinomial models. Multicollinearity among predictors was assessed using variance inflation factors (VIF), calculated within each imputed dataset and summarized across imputations; VIF values below 5 were considered indicative of acceptable collinearity. The adequacy of the sample size for multivariable modeling was evaluated using the events-per-variable (EPV) criterion, ensuring sufficient observations relative to the number of estimated parameters.

Statistical analysis was performed using the R software. Values of *p* < 0.05 were considered statistically significant.

## 3. Results

### 3.1. Profiles of Respondents

This study included 1155 participants (683 female, 472 male) with a mean age of 11.95 ± 3.31 years. Their key sociodemographic and health-related variables are summarized in Table 1.

### 3.2. Awareness of MNP Sources and Information Sources on MNPs

**Figure 1:**
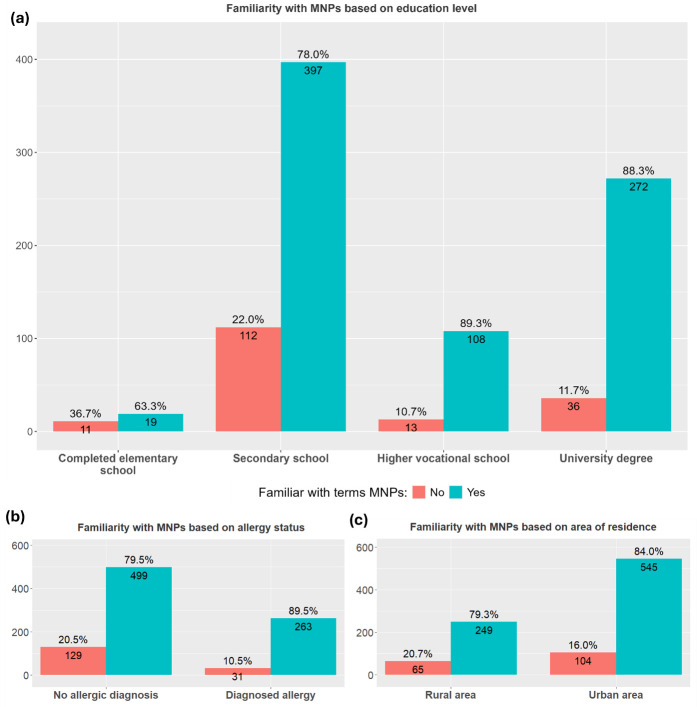
Awareness of micro- and nanoplastic (MNP) terminology. Percentage of participants who reported having heard of micro- and nanoplastics with total counts per response category: (**a**) educational level (elementary, secondary, higher vocational school or university degree), (*χ*^2^ = 25.461, *p* = 0.00001); (**b**) area of residence (urban or rural), (*χ*^2^ = 2.8827, *p* = 0.0895); and (**c**) allergy status (allergic or non-allergic), (*χ*^2^ = 13.266, *p* = 0. 0002702).

To estimate their familiarity with micro- and nanoplastics, participants were asked whether they had heard these terms before. The responses, segmented by area of residence, allergy status, and educational level, are presented in Figure 1.

The multivariate regression analysis, corrected for age, sex and SES, also revealed that higher vocational education was associated with increased familiarity with MNPs (OR = 3.17, 95% CI: 1.00–10.07, FDR *p* value = 0.050), as was the child having a diagnosis of allergy (OR = 1.78, 95% CI: 1.04–3.04, FDR *p* value = 0.036).

**Figure 2:**
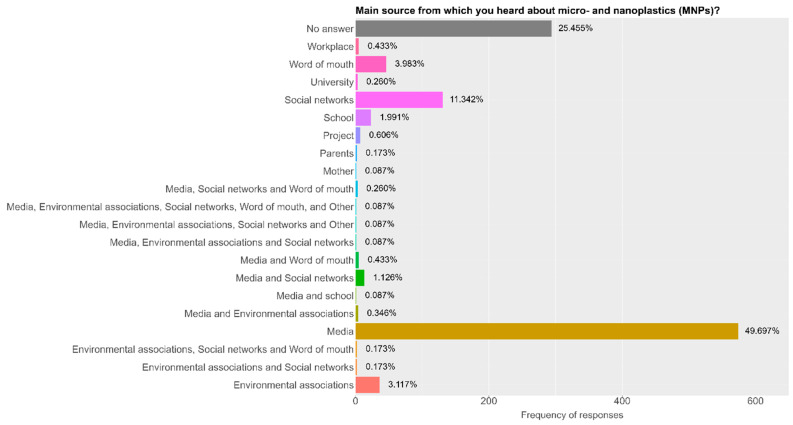
Information sources of micro- and nanoplastics.

Across all demographic groups, mass media and social media were identified as the primary sources of information about MNPs (Figure 2). The multivariant regression analysis, adjusted for age, sex and SES, confirmed that the source of information source was a significant influence: respondents obtaining information regarding MNPs from media and social media sources (OR = 18.67, 95% CI: 1.04–3.04, FDR *p* value = 0.026) or multiple sources (OR = 21.82, 95% CI: 1.21–393.33, FDR *p* value = 0.037) had higher odds of being familiar with MNPs. Although the wide confidence interval indicates variability in the magnitude of this effect, the positive trend remains statistically significant, suggesting a strong association between diverse information exposure and awareness.

### 3.3. Public Perception of MNP Contamination in Food

**Figure 3:**
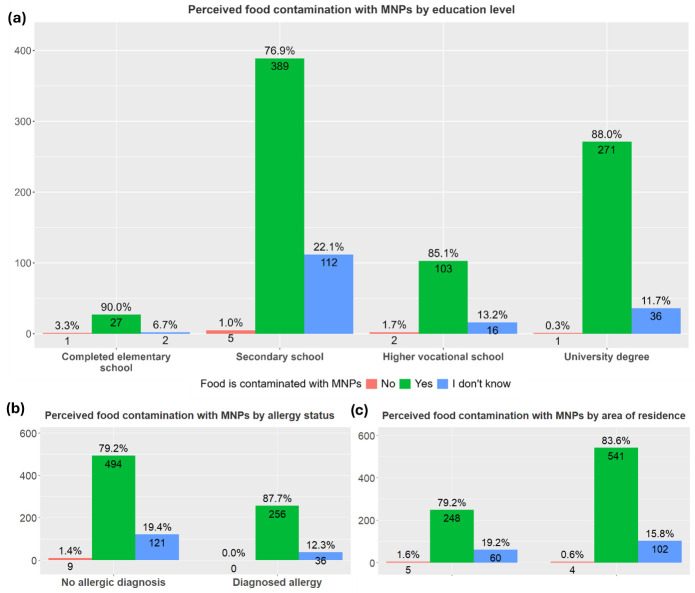
Perception about food contamination with micro- and nanoplastics (MNPs) across different categories based on (**a**) educational level (elementary, secondary, higher vocational school or university degree), (*χ*^2^ = 22.76, *p* = 0.0036); (**b**) allergy status (allergic or non-allergic), (*χ*^2^ = 11.757, *p* = 0.0028); (**c**) area of residence (urban or rural), (*χ*^2^ = 4.0994, *p* = 0.1266).

The majority of participants believed food products are contaminated with micro- and nanoplastics (69.35%). The type of residence was not found to affect participants’ views of food contamination with MNPs, whereas both a higher educational level and allergy status were associated with a significantly stronger conviction in this contamination, as seen in Figure 3.

The multivariate regression analysis (corrected for age, sex and SES) demonstrated that perception of food being contaminated with MNPs was positively associated with the child having a diagnosis of an allergic disease (OR = 1.95, 95% CI: 1.23–3.09, FDR *p* value = 0.005). In contrast, residing in the Mediterranean region of Dalmatia was associated with lower odds for perceived MNP food contamination (OR = 0.45, 95% CI: 0.26–0.77, FDR *p* value = 0.004).

### 3.4. Perceived Toxicity of MNP

**Figure 4:**
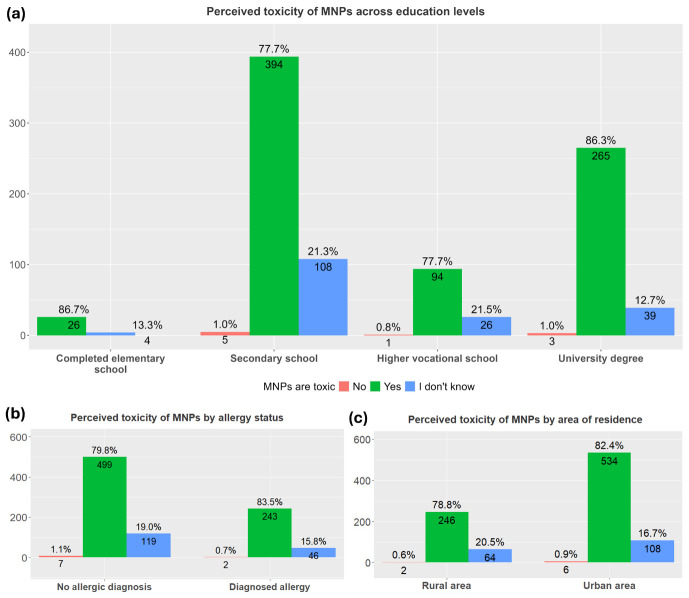
MNPs perceived as toxic among all studied groups. The percentage and total number of participants who perceived micro- and nanoplastics as toxic are presented per categories: (**a**) educational level (elementary, secondary, higher vocational school or university degree), (*χ*^2^ = 11.152, *p* = 0.0876); (**b**) area of residence (urban or rural), (*χ*^2^ = 2.2727, *p* = 0.3210); and (**c**) allergy status (allergic or non-allergic), (*χ*^2^ = 1.8594, *p* = 0.3947).

Participants were asked whether they agree that micro- and nanoplastics can be toxic or contain toxic materials. In total, 68.4% of participants perceived MNPs as toxic substances. This perception did not differ between participants of different places of residence, allergy status and level of education (Figure 4).

The multivariate regression analysis (corrected for age, sex and socioeconomic status) also did not reveal significant associations with the participants’ perception of MNP toxicity in food.

### 3.5. Opinions on Long-Term Health Effects of MNPs

**Figure 5:**
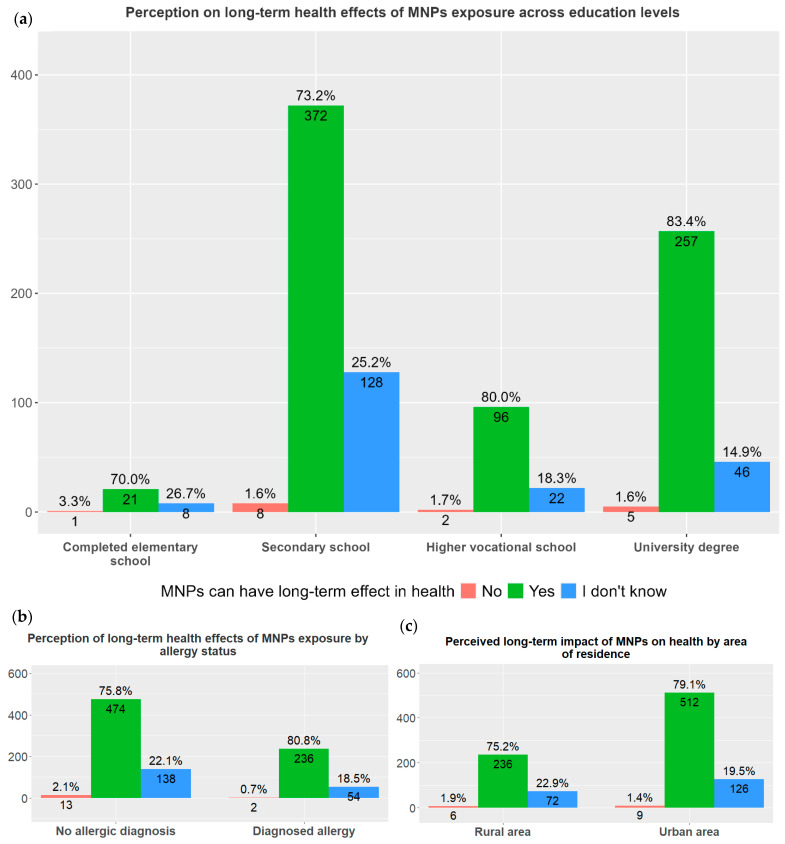
Public opinion on the long-term health effects of micro- and nanoplastics. Percentage of participants expressing opinions on the long-term health effects of micro- and nanoplastics, with total counts per response category: (**a**) educational level (elementary, secondary, higher vocational school or university degree), (*χ*^2^ = 13.902, *p* = 0.0308); (**b**) area of residence (urban or rural), (*χ*^2^ = 2.0202, *p* = 0.3642 ); and (**c**) allergy status (allergic or non-allergic), (*χ*^2^ = 4.2288, *p* = 0.1207).

The majority of the participants (82.6%) perceived MNP consumption to be associated with long-term health risks. Participants with higher levels of education (university degree) were more concerned about long-term health effects of MNPs (83.3%) compared to 70.0% of participants with elementary education (χ^2^ = 13.902, *p* = 0.0308). Participants’ opinions on this matter did not differ according to area of residence and allergy status (Figure 5).

However, the multivariate regression analysis (adjusted for age, sex and SES) did not reveal significant associations with the participants’ opinions on the potential long-term health effects of MNPs.

### 3.6. Recycling Engagement and Attitudes

**Figure 6:**
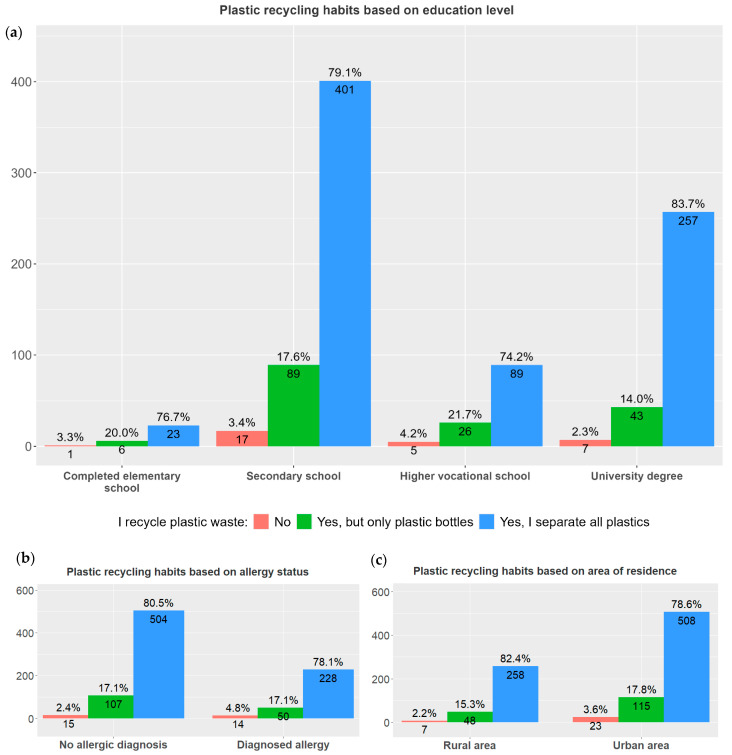
Participants attitudes towards plastic recycling. The figure presents the percentage of individuals reporting recycling of plastic bottles or all plastic waste with total counts per response category: (**a**) educational level (elementary, secondary, higher vocational school or university degree), (*χ*^2^ = 5.7476, *p* = 0.4520); (**b**) allergy status (allergic or non-allergic), (*χ*^2^ = 3.7731, *p* = 0.1516); and area of residence (urban or rural), (*χ*^2^ = 2.3152, *p* = 0.3142) (**c**).

The survey findings indicate that most respondents are aware and were inclined towards recycling plastic waste, regardless of their child’s allergy status (Figure 6 and Table 2).

## 4. Discussion

This large-scale cross-sectional study, which involved 1155 participants aged 6–18 years, investigated consumer perception and awareness of the health risks associated with micro- and nanoplastics (MNPs) and revealed associations with urban residence, education level, and allergy status. By focusing on vulnerable populations (children with allergic conditions) and key demographic factors, this study adds nuance to the growing body of research on public understanding and attitudes toward emerging environmental contaminants. Overall awareness of MNPs was high across all groups, consistent with previous reports [30,31,35,39,48]. Our results indicate that individuals residing in urban areas demonstrated greater awareness of potential health risks from MNPs compared with their rural counterparts. This difference may reflect greater access to information through mass and social media, broader educational opportunities, and potentially higher environmental exposure due to greater pollution levels in urban settings.

The rise in awareness is likely linked to increasing media coverage of microplastics in recent years [49]. In general, participants reported receiving information about MNPs mostly from social media platforms. Given the growing reliance on social and digital media for health and environmental information, these channels may serve as effective routes for future health campaigns aimed at raising risk awareness and promoting evidence-based understanding [30,47,48]. Similarly, Anderson et al. further emphasize the importance of visualization in awareness-building, noting that people are more likely to engage with issues they can readily see and imagine, making social media particularly powerful in shaping perceptions [39]. However, while this highlights social media’s influence in shaping public discourse, it also underscores the risk of misinformation due to the predominance of non-peer-reviewed content.

Notably, the majority of participants believed that food products are contaminated with MNPs, with allergic participants reporting the highest prevalence of this belief (87.7%), suggesting that allergy status is associated with awareness of environmental health risks. This may be explained by increased sensitivity toward potential triggers, as well as greater awareness of adverse health effects in this population. Biological plausibility supports this perception. In individuals with pre-existing allergic conditions, MNPs may increase allergen sensitivity by promoting inflammation, disturbing the epithelial barrier and gut microbiome, and acting as carriers for allergenic compounds [16,27,50]. Immunotoxicology studies reinforce these concerns, showing that exposure to polystyrene microplastics increases IgE-binding capacity and enhances Th2-mediated immune responses to house dust mite allergens in airway models [51]. Additionally, clinical findings also report higher levels of detected microplastics in individuals with allergic rhinitis, suggesting a possible link between allergy and microplastic accumulation in sensitive tissues [52].

Throughout our study, education consistently emerged as a factor influencing awareness of the health impacts of MNPs. Interestingly, participants with higher education levels expressed significant concern about long-term effects of MNP exposure. This suggests that higher education may equip individuals with the critical thinking skills and scientific literacy necessary to understand and interpret information about environmental health issues. This observation is supported by Rahman et al., who reported that education level had a strong positive effect on knowledge (β = 0.525, *p* < 0.01) and awareness (β = 0.057, *p* < 0.01). Similarly, Omoyajowo et al. identified a pronounced knowledge gap between individuals with lower and higher education, with 43.1% of respondents acknowledging limited understanding of microplastic pollution and its ecological impacts. These findings show that, while education fosters awareness, deeper knowledge of MNP-related risks remains insufficient even among more educated groups [53]. Intervention studies further demonstrate the value of education in shaping perceptions. For instance, Hogan et al. designed a curriculum for middle school students and found that participants significantly improved their ability to define microplastics and identify their sources following instruction, underscoring the importance of integrating this topic into formal education [54]. Likewise, a survey from 2020 in Shanghai revealed only 26% of respondents in Shanghai had heard of microplastics prior to participation, and 75% became worried once informed of the potential health impacts [34]. Together, these studies illustrate the importance of expanding public education, developing tailored curricula, and implementing nationwide awareness campaigns to strengthen both baseline knowledge and long-term understanding of microplastic-related health risks.

Beyond risk perception, this study also examined recycling behaviors and attitudes toward plastic waste management. Interestingly, statistical analysis revealed no significant differences in self-reported recycling habits across participants based on place of residence, allergy status, or education level, indicating a widespread adoption of recycling practices irrespective of demographic factors, seen also in EU reports for Attitudes of Europeans towards the environment [55]. This uniformity also suggests that recycling has become a well-established practice that transcends basic demographic boundaries, aligning with findings from the Special Eurobarometer 501 report, which documented similar pro-environmental behavioral patterns among European citizens regardless of their sociodemographic backgrounds [55]. However, it is important to note that self-reported data on pro-environmental behaviors may be subject to social desirability bias, with participants potentially overstating their engagement in recycling to align with socially valued norms. Such bias has been noted in prior environmental behavior research and should be considered when interpreting these findings [56]. Nevertheless, the support expressed for stricter regulations (83.3%) and for biodegradable alternatives (59%) suggests that, beyond possible overreporting, there is genuine public concern and willingness to engage in broader systemic changes to reduce plastic waste. Our results align with prior research, suggesting that recycling behavior is shaped more by established habits and the perceived convenience of recycling systems, rather than by education or urban–rural residence. These preferences again suggest that enhancing the convenience of recycling and providing incentives could further encourage pro-environmental behaviors [57]. As Hoang points out, shifting public behavior and reducing plastic use demands a systematic approach involving manufacturers, users, and waste managers [58]. The lower tendencies toward plastic recycling behavior in the Mediterranean region is noteworthy and may point out regional differences and lifestyle habits between Croatian regions [59]. However, since the least number of participants were involved in this region, this finding may likely be due to the disparity in the sample sizes between subgroups of the participants according to region of origin.

Overall, this study offers valuable insights into factors shaping public perception and awareness of MNP-related health risks. With asthma and allergies increasingly becoming more prevalent among children in recent decades, these vulnerable populations deserve particular attention in future research. Our results may guide the development of targeted risk communication strategies aimed at improving understanding and encouraging informed decision-making. Future research and public health efforts should focus on creating effective ways to address misunderstandings about MNP exposure. It is important to empower people to make informed decisions. Raising awareness through educational programs and including the topic of microplastics in school curricula will be crucial for building understanding. Additionally, given the central role of social media in shaping public perceptions, these platforms should be leveraged to disseminate accurate, evidence-based information while actively countering misinformation. Finally, future work should examine the links between knowledge, attitudes, and behavior, especially in certain populations, such as patients with chronic conditions and multi-morbid allergic phenotypes, as this will be essential for developing interventions that not only raise awareness but also foster meaningful behavior change to protect both human health and the environment.

### Study Limitations

The present study has certain limitations that should be acknowledged. First, due to the cross-sectional nature of the study design, it is not possible to establish causality between the variables. Specifically, we cannot determine whether a pre-existing allergy diagnosis leads to a heightened perception of health risks from MNPs, or if individuals with naturally higher risk perception are more likely to seek medical consultation and thus receive an allergy diagnosis. Future longitudinal studies would be required to clarify these temporal relationships. Secondly, this study primarily captures perceived awareness rather than objective knowledge. As these measures rely on self-reported data, there is a possibility the awareness levels are overestimated due to social desirability or subjective interpretation of knowledge. This distinction between perceived and actual knowledge should be considered when interpreting the results and should be further addressed in future studies. Moreover, participants with pre-existing conditions (such as allergy) might have been more keen to participate in this study and more cooperative to provide data on their perception of MNPs, compared to their non-allergic peers, yielding in overestimation of the higher proportion of participants with allergies that have increased awareness on the issue. However, the large sample size (>900 participants) and the less than 1:3 ratio in allergic respondents vs. their non-allergic peers should account for this potential bias. There was also a notable difference in the sample size of participant subgroups according to the region of origin, which may have contributed to bias. Furthermore, the assessment of risk perception relied mostly on binary items (yes or no), and the lack of a multi-item Likert scale limits the assessment of internal consistency and may affect the construct’s depth. However, while limiting depth, binary items were adopted to ensure a quick and straightforward assessment of risk perception, reducing cognitive load for the participants and limiting ambiguous responses. Nevertheless, future studies are required to address this issue using validated multi-item Likert scales, as well as other options. Another limitation of this study is also the high rate of missing data for certain variables. Although this may introduce a degree of non-response bias, the missing data was imputed based on variable type in the multivariant regression analysis. However, even though multiple imputation was applied to address missing data, certain limitations remain. The proportion of missing data for key variables should be considered when evaluating the robustness of the findings. Furthermore, since the missingness is not completely random, this may introduce bias that is not fully mitigated by the imputation approach. Although potential confounders such age, sex and socioeconomic status of the participants were included in the adjusted multivariate regression analysis, other factors beyond these common covariates, such as parental attitudes, health literacy, media exposure intensity, etc., may have influenced the results of this study. Future studies should incorporate a broader range of psychosocial and behavioral variables—such as parental/caregiver/family attitudes and awareness, their health literacy, etc.—through validated instruments and, where possible, longitudinal or mixed-method designs to better account for residual confounding and strengthen causal inference. The fact that most of the participants provided data by proxy (having their parents/caregivers fill out the questionnaire) represents an important methodological consideration. This may have introduced discrepancies between the child’s actual perceptions and the reported responses, as caregivers may interpret or estimate children’s awareness differently. Consequently, proxy reporting may have led to systematic bias in the assessment of the main outcomes, particularly in measures related to subjective perceptions. Additionally, this may hinder the true level of awareness as the parents/caregivers might have been less inclined to reveal their attitudes and habits in fear of judgment for being less invested in their child’s best interests. Finally, the fact that this study included participants from a single national setting, with its own unique sum of mentality, lifestyle habits and other determinants of public opinion, likely means that these results are population-specific. Therefore, future, well-structured, larger studies involving multiple different populations assessed and followed-up for a longer period of time are required to confirm these results and adequately address the issue of true consumer perception and awareness of MNPs.

## 5. Conclusions

This study reveals high awareness of micro- and nanoplastics (MNPs) among the Croatian population, with place of residence, education level, and allergy status significantly shaping risk perception. Urban and higher-educated participants showed greater concern, while allergic individuals were particularly sensitive to MNP-related risks. The greatest difference was observed between allergic and non-allergic participants regarding concerns about MNP contamination in food, indicating that allergic individuals are more aware of environmental and health risks due to their heightened vigilance and prior experiences with adverse reactions. By focusing on allergic individuals, a vulnerable and growing population, this study fills a key research gap and lays the foundation for future investigations on MNP exposure and chronic disease. Social media was the primary source of information, highlighting both its outreach potential and risk of misinformation. Strong public support for stricter regulations and biodegradable alternatives indicates readiness for systemic change, while education emerged as the main driver of informed attitudes and sustainable behavior. Targeted health communication is needed to reach vulnerable groups and populations with lower awareness of MNP-related risks. Strengthening education, ensuring reliable information on social media, and implementing public campaigns could improve understanding, encourage protective behaviors, and support policies to reduce plastic pollution.

The direction of causality in factors contributing to individual perception and awareness of MNPs remains to be explored in future longitudinal studies, involving multiple cohorts, including patients with chronic conditions and specific disease phenotypes.

## Figures and Tables

**Table 1:** Sociodemographic and health-related variables of respondents (*N* = sample size).

Variables	*n* *	%
**Type of residence area (*N* = 964):**		
Urban area	650	67.4
Rural area	314	32.6
Region of origin (Zagreb region)	271	23.5
Region of origin (Dalmatia region)	254	22.0
Region of origin (Slavonia region)	627	54.3
**Live nearby major roads (*N* = 947):**		
Yes	593	62.6
No	354	37.4
**Own a garden nearby major road (*N* = 924):**		
Yes	122	13.2
No	802	86.8
**Number of adults living in household (*N* = 966):**		
One	50	5.2
Two	642	66.4
Three	177	18.3
Four or more	97	10.1
**Number of children living in household (*N* = 942):**		
One	214	22.7
Two	468	49.7
Three	203	21.5
Four or more	42	6.1
**Monthly income of household (*N* = 917):**		
<600 euros	61	6.7
600–1300 euros	289	31.5
1300–2000 euros	291	31.7
>2000 euros	276	30.1
**Education level of parents (*N* = 969):**		
Elementary	30	3.1
Secondary	121	12.5
Higher vocational school	309	31.9
University degree	509	52.5
**Children’s allergy status (*N* = 934):**		
None	637	68.2
Allergic **	297	31.8
Allergic asthma	70	23.6
Allergic rhinitis	93	31.3
Atopic dermatitis	114	38.4
Food allergy	62	20.9

* The sample size (*N*) varies across categories because not all respondents answered every question. Percentages were calculated based on valid responses only, excluding missing data from the denominator for each specific variable. ** The total percentage for specific allergic diseases (e.g., allergic asthma, rhinitis, etc.) may exceed 100% because respondents were permitted to select multiple conditions if the child suffers from more than one allergy.

**Table 2:** Public practice and attitudes toward plastic waste reduction, recycling, and policy measures (*n* indicates sample size per question, CI indicates confidence interval).

Category and Question	*n* *	Yes (%)	95% CI
**I. Behavioral and Factual Data**			
Is there separate sorting of plastic waste in your city/town?	952	82.5	80.08–84.92
When using your own bags, are they made of plastic?	969	30.0	27.12–32.88
**II. Public Attitudes and Opinions**			
Would you prefer that your local authorities implement stricter measures to improve the quality of plastic waste recycling?	975	75.5	72.80–78.20
If offered stimulus, will that encourage you to recycle?	952	75.8	73.08–78.52
Do you agree that reducing single-use plastics could help reduce plastic waste?	978	83.3	80.96–85.64
Do you think that charging single-use plastic bags can effectively reduce plastic waste?	977	51.0	47.87–54.13
Would you be willing to pay more for biodegradable bags or products with biodegradable packaging?	972	59.0	55.91–62.09
Would you like plastic bags and plastic packaging to be completely removed from stores?	976	69.0	66.10–71.90
Do you think the use of microplastics in cosmetics should be prohibited?	971	80.0	77.48–82.52

* *n* = number of respondents for each specific question; 95% CI = 95% confidence interval for the proportion of “Yes” responses. The multivariate regression analysis, adjusted for age, sex and SES, showed that plastic recycling behavior was lower among respondents residing in the Dalmatia region (OR = 0.14, 95% CI: 0.04–0.44, FDR *p* value < 0.001).

## Data Availability

The data presented in this study are available on request from the corresponding author, under specific conditions. The data is not publicly available due to ethical restrictions (sensitive data).

## Supplementary Materials

The following supporting information can be downloaded at: https://www.mdpi.com/article/10.3390/children13040470/s1, Figure S1: Ethics committee approvals for the IMPTOX study in Croatian. IMPTOX—Horizon 2020; Figure S2: Ethics committee approvals for the IMPTOX study—translation to English; Figure S3: Ethics committee approvals for the EDIAQI study in Croatian; Figure S4: Ethics committee approvals for the EDIAQI study—translation to English; Table S1: Full list of allergens used in skin prick testing of participants; Table S2: MNP awareness among participants.
