# Supplementary material for: Perceiving the Invisible Threat: Are Allergic Individuals Aware of the Health Risks of Micro- and Nanoplastics?
Source: Children (Basel). 2026 Mar 28;13(4):470. doi: 10.3390/children13040470 (PMC13115234; doi:10.3390/children13040470)
Supplement: Supplementary file 1 [file children-13-00470-s001.zip › children-4174773-supplementary.pdf]

Supplementary Materials

Perceiving the Invisible Threat: Are Allergic Individuals Aware of the Health Risks of Micro- and Nanoplastics?

Ana Kujavec 1, Manuela Oroz 1, Jan Pantlik 1, Ivana Banić 1,2,*, Sandra Mijač 1, Ana Vukić 1, Petra Anić 1, Ana-Marija Genc 1, Antonija Piškor 1, Maja Šutić 1, Marcel Lipej 3, Željka Vlašić Lončarić 4, Milan Jurić 5, Ivana Marić 5, Vlatka Drinković 5, Tin Kušan 5, Rajka Lulić Jurjević 6,7 and Mirjana Turkalj 5,7,8,*

1 Department of Medical Research, Srebrnjak Children's Hospital, HR-10000 Zagreb, Croatia
2 Department of Innovative Diagnostics, Srebrnjak Children's Hospital, HR-10000 Zagreb, Croatia
3 IT Department, Srebrnjak Children's Hospital, HR-10000 Zagreb, Croatia
4 Department of Pulmonology, Srebrnjak Children's Hospital, HR-10000 Zagreb, Croatia
5 Department of Allergy and Clinical Immunology, Srebrnjak Children's Hospital, HR-10000 Zagreb, Croatia
6 Department of Cardiology, Srebrnjak Children's Hospital, HR-10000 Zagreb, Croatia
7 Faculty of Medicine, J.J. Strossmayer University of Osijek, HR-31000 Osijek, Croatia
8 Faculty of Medicine, Catholic University of Croatia, HR-10000 Zagreb, Croatia
* Correspondence: ibanic@bolnica-srebrnjak.hr (I.B.); mturkalj@bolnica-srebrnjak.hr (M.T.)

SREBRNJAK

Dječja bolnica Srebrnjak
Srebrnjak 100, Zagreb
Tel: 01 6391 100
Zagreb, 19.10.2021.
KLASA: 100-02/21-01
Ur.broj: 04-930/3-21

Članovi Etičkog povjerenstva su na 11. sjednici održanoj dana 19.10.2021. godine, s četiri (4) glasa ZA donijeli slijedeću

ODLUKU

Članak 1.

Odobrava se provođenje znanstveno istraživačkog projekta pod nazivom: EU H2020 ImpTox („ An innovative analytical platform to investigate the effect and toxicity of micro and nano plastics combined with environmental contaminants on the risk of allergic diseases in preclinical and clinical studies“), voditelja projekta izv.prof.dr.sc. Mirjane Turkalj, dr.med..

Članak 2.

Ova odluka stupa na snagu danom donošenja.

Obrazloženje

Dr.sc. Ivana Banić, mag.mol.biol. predala je Etičkom povjerenstvu Dječje bolnice Srebrnjak dana 30.09.2021.g. Zamolbu za odobrenje znanstveno istraživačkog projekta pod nazivom EU H2020 ImpTox financiranog sredstvima Europske unije za istraživanje i inovacije u sklopu okvirnog programa Obzor 2020 (Horizon 2020), Grant agreement number: 965173.
Cilj projekta je detaljnije istraživanje utjecaja mikro i nanoplastike iz okoliša, osobito u kombinaciji s drugim okolišnim onečišćivačima na ljudsko zdravlje s naglaskom na razvoj i kliničke manifestacije alergijske bolesti kod djece. Istraživanje će uključivati djecu dobi od 6 do 18 godina na nacionalnoj razini, u školama, kroz tri glavne geografske regije. Projekt je na sjednici Etičkog povjerenstva predstavio istraživač na projektu prof.dr.sc. Davor Plavec, dr.med. Članovi povjerenstva su sa 4 glasa ZA, odobrili provođenje istraživačkog projekta te je valjalo odlučiti kao u izreci Odluke.

Predsjednica Etičkog povjerenstvo
doc.dr.sc. Helena Munivrana Škvorc, dr.med.

Doc.dr.sc. Helena Munivrana Škvorc, dr.med.
specijalist pedijatar
alergolog i kl. imunolog
0785765

Dostaviti:
1. Podnositelju zamolbe
2. Arhiva

QUALITY SYSTEM CERTIFICATION
DNV·GL
ISO 9001:2015

Figure S1. Ethics committee approvals for the IMPTOX study, original in Croatian. Horizon 2020 - IMPTOX (An innovative analytical platform to investigate the effect and toxicity of micro and nanoplastics combined with environmental contaminants on the risk of allergic disease in preclinical and clinical studies, grant agreement number: 965173).

Srebrnjak Children`s Hospital
Srebrnjak 100,
HR-10000 Zagreb
Croatia
Tel: +385 1 6391 100
Zagreb, 19th October 2021

CLASS: 100-02/21-01
Rec.No.: 04-930/3-21

On 19th October 2021 at the 11th session of the Ethics Committee of Srebrnjak Children`s Hospital, with 4 votes PRO the members of the Ethics Committee have reached the following

DECISION

Article 1.

The implementation of the research project and the clinical study within the ***EU Horizon 2020 IMPTOX ("An innovative analytical platform to investigate the effect and toxicty of micro- and nano plastics combined with environmental contaminants on the risk of allergic diseases in preclinical and clinical studies"),*** Principal Investigator: prof. Mirjana Turkalj, MD, PhD has been APPROVED hereby.

Article 2.

This decision becomes final on the day it was reached.

Elaboration

On 30th September 2021, Ivana Banić, PhD, MSc in Mol Biol has submitted the request for approval of the research project EU Horizon 2020 IMPTOX ("An innovative analytical platform to investigate the effect and toxicty of micro- and nano plastics combined with environmental contaminants on the risk of allergic diseases in preclinical and clinical studies"), funded by the EU Horizon framework programme for research and innovation, Grant agreement ID: 965173, along with relevant ethics related documentation.
The aim of this research project is to investigate the effects of environmental micro- and nanoplastics, especially in combination with other environmental pollutants, on human health, with a special focus on the development and clinical manifestations of allergic diseases in children. The study will involve children aged 6 to 18 years in schools nation-wide, in 3 main geographical regions in Croatia. The project research was presented by prof. Davor Plavec, MD, PhD, at this Ethics Committee session. The members of the Ethics Committee have approved the implementation of this research with 4 votes PRO, reaching the afore mentioned Decision.

The President of the Ethics Committee of the Srebrnjak Children`s Hospital
Assoc. Prof. Prim. Helena Munivrana Škvorc, MD, PhD

Deliver to:
1. The applicant
2. Archives

Figure S2. Ethics committee approvals for the IMPTOX study- translation to English. Horizon 2020 - IMPTOX (An innovative analytical platform to investigate the effect and toxicity of micro and nano plastics combined with environmental contaminants on the risk of allergic disease in preclinical and clinical studies, grant agreement number: 965173).

SREBRNJAK

Dječja bolnica Srebrnjak
Srebrnjak 100, Zagreb
Tel: 01 6391 100
Zagreb, 01.02.2023.
KLASA: 100-02/23-01
Ur.broj: 04-100/3-23

Članovi Etičkog povjerenstva su na 20. sjednici održanoj dana 01.02.2023. godine, sa pet glasova ZA donijeli slijedeću

ODLUKU

Članak 1.

Odobrava se provedba znanstveno-istraživačkog projekta pod nazivom ***EU H2020 EDIAQI („Evidence Driven Indoor Air Quality Improvement"),*** voditelj projekta prof.dr.sc. Mirjana Turkalj, dr.med.

Članak 2.

Ova odluka stupa na snagu danom donošenja.

Obrazloženje

Dr.sc. Ivana Banić, mag.mol.biol. podnijela je Etičkom povjerenstvu Zamolbu za odobrenje provedbe znanstveno-istraživačkog projekta pod nazivom EU H2020 EDIAQI („Evidence Driven Indoor Air Quality Improvement"), financiranog sredstvima EU za istraživanje i inovacije u sklopu okvirnog programa Obzor 2020 (Horizon 2020), Grant agreement ID: 101057497. Voditelj projekta je prof.dr.sc. Mirjana Turkalj, dr.med..
Sukladno podnesenoj Zamolbi, članovi povjerenstva su na 20. elektronskoj sjednici dana 01.02.2023. ocijenili provedbu znanstvenog istraživanja etički prihvatljivim, te je donesena Odluka kao u Izreci.

Predsjednica Etičkog povjerenstvo
prim.doc.dr.sc. Helena Munivrana Škvorc, dr.med.

Doc. prim. dr. sc.
Helena Munivrana Škvorc, dr. med.
specijalist pedijatar
alergolog i klinički imunolog
185765

Dostaviti:
1. Podnositelju zamolbe
2. Arhiva

Figure S3. Ethics committee approval for the EDIAQI study, original in Croatian. Horizon Europe EDIAQI (Evidence driven indoor air quality improvement, grant agreement number 101057497).

On 1st February 2023 at the 20th Ethics Committee session, with 5 votes PRO the members of the Ethics Committee have reached the following

DECISION

Article 1.

The implementation of the research project and the clinical study within the ***EU Horizon 2020 EDIAQI ("Evidence Driven indoor Air Quality Improvement")***, Principal Investigator: Prof. Mirjana Turkalj, MD, PhD has been APPROVED hereby.

Article 2.

This decision becomes final on the day it is reached.

Elaboration

Ivana Banić, PhD, MSc in Mol Biol has submitted the request for approval of the research project EU Horizon 2020 ("Evidence Driven Indoor Air Quality Improvement"), funded by the EU Horizon framework programme for research and innovation, Grant agreement ID: 101057497, along with relevant ethics related documentation. The Principal Investigator of the study is Prof. Mirjana Turkalj, MD, PhD.

According to this request, the members of the Ethics Committee of the Srebrnjak Children`s Hospital at the 20th Ethics Committee session held online on 1st February 2023 have found this study and the research project valid and ethically acceptable, thus reaching the afore mentioned Decision.

The President of the Ethics Committee of the SRebrnjak Children`s Hospital

Assoc. Prof. Prim. Helena Munivrana Škvorc, MD, PhD

Deliver to:

1. The applicant

2. Archives

Figure S4. Ethics committee approvals for the EDIAQI study- translation to English. Horizon Europe EDIAQI (Evidence driven indoor air quality improvement, grant agreement number: 101057497).

Table S1. Full list of allergens used in skin prick testing of participants.

Type of allergen	Allergen species	Binomial nomenclature
**House dust**	House dust mite	*Dermatophagoides farinae*<br>*Dermatophagoides pteronyssinus*
**Animal dander**	Cat dander	*Felis domesticus*
	Dog dander	*Canis familiaris*
**Grass pollen**	5 grasses mix- cocksfoot, sweet vernal-grass, rye-grass, meadow grass, and timothy	*Dactylis glomerata, Anthoxanthum odoratum, Lolium perenne, Poa pratensis and Phleum pratense*
**Weed pollen**	Common ragweed	*Ambrosia elatior*
	Mugwort	*Artemisia vulgaris*
	Lichwort	*Parietaria officinalis*
**Tree pollen**	Common silver birch	*Betula verrucosa*
	Hazel	*Corylus avellana*
	Olive	*Olea europaea*
	Pine	*Pinus radiata*
	Cypress	*Cupressus sempervirens*
**Shrub pollen**	Mimosa	*Mimosa pudica*
**Molds**	Alternaria	*Alternaria alternata*
	Cladosporium	*Cladosporium herbarum or spp.*
**Food allergens**	Whole egg	*Gallus spp.*
	Cow`s milk	*Bos spp.*
	Wheat flour	*Triticum aestivum*
	Corn flour	*Zea mays*
	Soy	*Glycine max (Soja hispida)*
	Peanut	*Arachis hypogaea*
	Sesame	*Sesamum indicum*
	Hake	*Merluccius merluccius*
	Trout	*Salmo trutta fario*
	Hazelnut	*Corylus avellana*
	Walnut	*Juglans spp.*
	Almond	*Amygdalus communis*
	Cocoa	*Theobroma cacao*
	Rice	*Oryza sativa*

Table S2. Questionnaire on Socioeconomic Status, Lifestyle Habits, and MNP Awareness Among Participants.

Question	Possible answers
**1. Have you ever heard of the terms "microplastics" and "nanoplastics"?**	a) Yes<br>b) No
**2. What is the main source from which you have heard about micro-/nanoplastics?**	a) Media (TV, radio, websites)<br>b) Environmental associations or campaigns<br>c) Social media<br>d) Orally (friends or acquaintances)<br>e) Other:__________________________
**3. Do you know the sources of micro- and nanoplastics?**	a) Yes<br>b) No
**4. In your opinion, where could micro- and nanoplastics (MNPs) be found?**	a) Yes<br>b) No
**5. Do you use products containing microbeads (e.g., toothpaste, exfoliating gels, soaps, laundry detergent)?**	a) Yes<br>b) No<br>c) I do not know
**6. How many single-use plastic bags do you typically take when shopping?**	a) One<br>b) More than one<br>c) I do not buy plastic bags but use environmentally friendly alternatives instead (paper, biodegradable, or cloth bags)<br>d) I usually bring my own bags
**7. When using your own bags, are they plastic?**	a) Mostly yes<br>b) Mostly not
**8. Would you like plastic bags and plastic packaging to be completely removed from stores?**	a) Yes<br>b) No
**9. Would you be willing to pay more for biodegradable bags or products with biodegradable packaging?**	a) Yes<br>b) No
**10. Do you think that the food you consume might be contaminated with microplastics?**	a) Yes

	b) No<br>c) I do not know
**11. Do you think micro- and nanoplastics might be toxic or contain toxic materials?**	a) Yes<br>b) No<br>c) I do not know
**12. Do you think microplastics could have a long-term effect on your health?**	a) Yes<br>b) No<br>c) I do not know
**13. Is there separate sorting of plastic waste in your city/town?**	a) Yes, there are separate containers for plastic waste<br>b) There are only containers for plastic bottle collection<br>c) No, there is no system for separate sorting of plastic waste
**14. Do you recycle your plastic waste?**	a) Yes, I separate plastic waste into separate containers<br>b) Yes, but only plastic bottles<br>c) No
**15. If offered stimulus, will that encourage you to recycle?**	a) Yes<br>b) No
**16. Do you agree that reducing single-use plastics could help reduce plastic waste?**	a) I agree<br>b) I disagree
**17. Do you think the use of microplastics in cosmetics should be prohibited?**	a) I agree<br>b) I disagree
**18. Do you think that charging single-use plastic bags can effectively reduce plastic waste?**	a) Yes<br>b) No
**19. Would you prefer that your local authorities implement stricter measures to improve the quality of plastic waste recycling?**	a) Yes<br>b) No
**20. What is your sex?**	a) Female<br>b) Male

**21. Parental/caregiver status:**	a) Employed<br>b) Works in his/her own company<br>c) Unemployed, looking for job<br>d) Unemployed due to health condition<br>e) Works from home, in agriculture<br>f) Student<br>g) Retired<br>h) Other
**22. What is the education background of you/your parents?**	a) Elementary school (<10-12 years)<br>b) Secondary school (10-12 years)<br>c) Higher vocational school<br>d) University degree
**23. What is the name of your city/village you live in?**	
**24. In what area do you live in?**	a) Urban<br>b) Rural (population <5000)
**25. If you live in urban area, what is the population of your city?**	
**26. If you live in rural area, do you reside on farmstead?**	a) Yes<br>b) No
**27. Do you live nearby major roads?**	a) Yes<br>b) No
**28. Do you own a garden/orchard near major roads?**	a) Yes<br>b) No
**29. What is the monthly income of your household (including salaries, sale of agricultural products, rents and other income sources)?** *Note: 1 Croatian kuna (HRK) ≈ 0.13 euros (EUR).*	a) up to 5000 HRK<br>b) 5000 – 10000 HRK<br>c) 10000 – 15000 HRK<br>d) more than 15000 HRK
**30. How many people live in your household?**	a) Number of adults: ___________<br>b) Number of children (<18 years): __________
